# Cryomicroneedle Arrays for Biotherapeutics Delivery

**DOI:** 10.1002/smsc.202500009

**Published:** 2025-06-08

**Authors:** Chunli Yang, Li Zhang, Angxi Zhou, Siyi Wang, Ya Ren, Maya Xiang, Run Tian, Yang Yu, Rong Li, Maling Gou

**Affiliations:** ^1^ Department of Biotherapy Cancer Center and State Key Laboratory of Biotherapy West China Hospital Sichuan University Chengdu Sichuan Province 610041 China; ^2^ Department of Head and Neck Oncology Cancer Center and State Key Laboratory of Biotherapy West China Hospital Sichuan University Chengdu Sichuan Province 610041 China; ^3^ Huahang Microcreate Technology Co., Ltd Chengdu Sichuan Province 610041 China; ^4^ Department of Chemistry University of Washington‐Seattle Campus Seattle WA 98195 USA; ^5^ Department of Dermatology West China Hospital Sichuan University Chengdu Sichuan Province 610041 China; ^6^ Department of Thoracic Oncology Cancer Center and State Key Laboratory of Biotherapy West China Hospital Sichuan University Chengdu Sichuan Province 610041 China; ^7^ Antibiotics Research and Re‐evaluation Key Laboratory of Sichuan Province Sichuan Industrial Institute of Antibiotics School of Pharmacy Chengdu University Chengdu Sichuan Province 610041 China

**Keywords:** biotherapeutics, cryomicroneedles, diseases, drug delivery, treatment

## Abstract

Biotherapy offers a promising approach for treating a variety of diseases. However, the lack of advanced delivery systems remains a significant barrier to improve the efficacy, safety, and cost‐effectiveness of biotherapeutics. The microneedle, as a minimally invasive drug delivery tool, has demonstrated considerable potential in biotherapeutic applications. Despite this promise, challenges remain in fabricating microneedles that effectively preserve the bioactivity of biotherapeutics. Emerging as a novel solution, cryomicroneedles (cryoMNs) employ cryogenically molded ice matrices that exploit phase‐transition thermodynamics. The metabolic stasis induced by cryoimmobilization preserves biomolecular conformation and cellular viability. Moreover, the ice‐reinforced architectures achieve an optimal balance between mechanical penetration capacity and post‐insertion dissolution kinetics, overcoming the rigidity‐flexibility trade‐off in traditional dissolving microneedles. Current research prioritizes three breakthrough directions: material innovation for cryocompatible polymer‐ice interfaces, cold‐chain optimization strategies to enhance payload viability, and innovations in medical application scenarios. Notably, preclinical successes in regenerative tissue engineering and thermostable vaccine platforms highlight cryoMNs’ potential to bridge precision medicine and global health equity. This review provides an overview of recent advancements in cryoMNs and discusses the potential challenges and future directions for the development of cryoMNs‐mediated biotherapeutics delivery.

## Graphical abstract

CryoMNs for biotherapeutic delivery to treat various diseases. CryoMNs: cryomicroneedles.

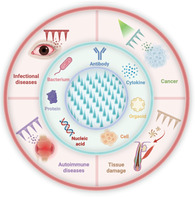

## 1 Introduction

Biotherapeutics are drug therapy products derived or produced from biological sources. These include recombinant proteins and hormones, monoclonal antibodies, cytokines, growth factors, gene therapy products, vaccines, cell‐based products, gene‐silencing/editing therapies, tissue‐engineered products, and stem cell therapies.^[^ ^1^, ^2^, ^3^, ^4^, ^5^, ^6^, ^7^ ^]^ Biotherapeutics offer new avenues for the treatment of challenging diseases, including cancers,^[^ ^8^ ^]^ infection diseases,^[^ ^9^ ^]^ autoimmune diseases,^[^ ^10^ ^]^ and tissue regeneration.^[^ ^11^ ^]^ However, oral administration of biotherapeutics is limited due to the disadvantages associated with digestive enzymes, acidic conditions, and first‐pass metabolism,^[^ ^12^ ^]^ which significantly reduces their bioavailability. Thus, selecting an appropriate drug delivery route is essential for improving drug bioavailability, enhancing efficacy, and minimizing toxic side effects. Transdermal drug delivery, as a crucial method of biotherapeutics administration, exhibits significant advantages in multiple aspects.^[^ ^13^, ^14^, ^15^ ^]^ It avoids gastrointestinal degradation and the first‐pass effect, thereby increasing bioavailability and ensuring consistent treatment effectiveness.^[^ ^16^, ^17^ ^]^

A microneedle array (MNA) is a patch containing micron‐scale needles (typically several hundred micrometers in length) capable of penetrating the skin barrier.^[^ ^18^, ^19^, ^20^ ^]^ This technology offers a minimally invasive approach for localized delivery of biotherapeutics, effectively circumventing the limitations of oral drug administration while avoiding injection‐associated discomfort. MNA can encapsulate biotherapeutics either within the needles or on their surface.^[^ ^21^, ^22^ ^]^ Alternatively, they can deliver drugs through microchannels formed by skin or cornea penetration.^[^ ^23^, ^24^ ^]^ According to drug delivery characteristics, microneedles (MNs) can be broadly classified into five types: solid MNs, hollow MNs, coated MNs, dissolving MNs, and porous MNs.^[^ ^19^ ^]^ Each type has distinct advantages and disadvantages. Solid, hollow, and coated MNs are mainly made from non‐biodegradable materials, such as silicon or metal, which provide good mechanical strength. However, they carry a potential risk of leaving residual broken needles in the body. Moreover, conventional MNs often have low drug permeation rates and may not preserve biotherapeutics’ activity over extended periods.

**Figure 1:**
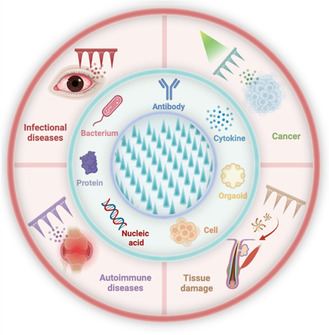
CryoMNs for biotherapeutic delivery to treat various diseases.

Cryomicroneedles (cryoMNs), also known as ice MNs, are a novel type of dissolving MNs fabricated from liquid‐containing materials and molded in a frozen state. CryoMNs inherit the advantages of dissolving MNs, while offering enhanced drug‐loading capacity by allowing MNs to be composed entirely of the therapeutic agent.^[^ ^25^ ^]^ Furthermore, cryopreservation reduces the metabolic rate of living cells or bacteria, by exploiting the temperature‐dependent exponential decay of biochemical reaction rates that occurs below freezing thresholds. This characteristic proves particularly advantageous in maintaining the structural integrity of bioactive compounds while prolonging their storage stability.^[^ ^26^, ^27^ ^]^ Additionally, the frozen state can increase the hardness of water‐containing microneedle materials,^[^ ^28^ ^]^ thereby improving their penetration capability. These features enable cryoMNs to pre‐load biotherapeutics and show potential for clinical applications across various scenarios, offering a promising new strategy for disease prevention and treatment (**Figure** **1**).

In this review, we present recent advancements in cryoMNs, with a specific focus on their design, fabrication, and applications in cancers, infectious diseases, autoimmune diseases, and tissue regeneration. We also discuss key characteristics of cryoMNs, including fabrication methods, materials, delivery sites, and functions. Finally, we address potential challenges and provide insights into the future development of cryoMNs‐mediated biotherapeutics.

## 2 CryoMNs Focus

### 2.1 Design and Fabrication of CryoMNs

**Figure 2:**
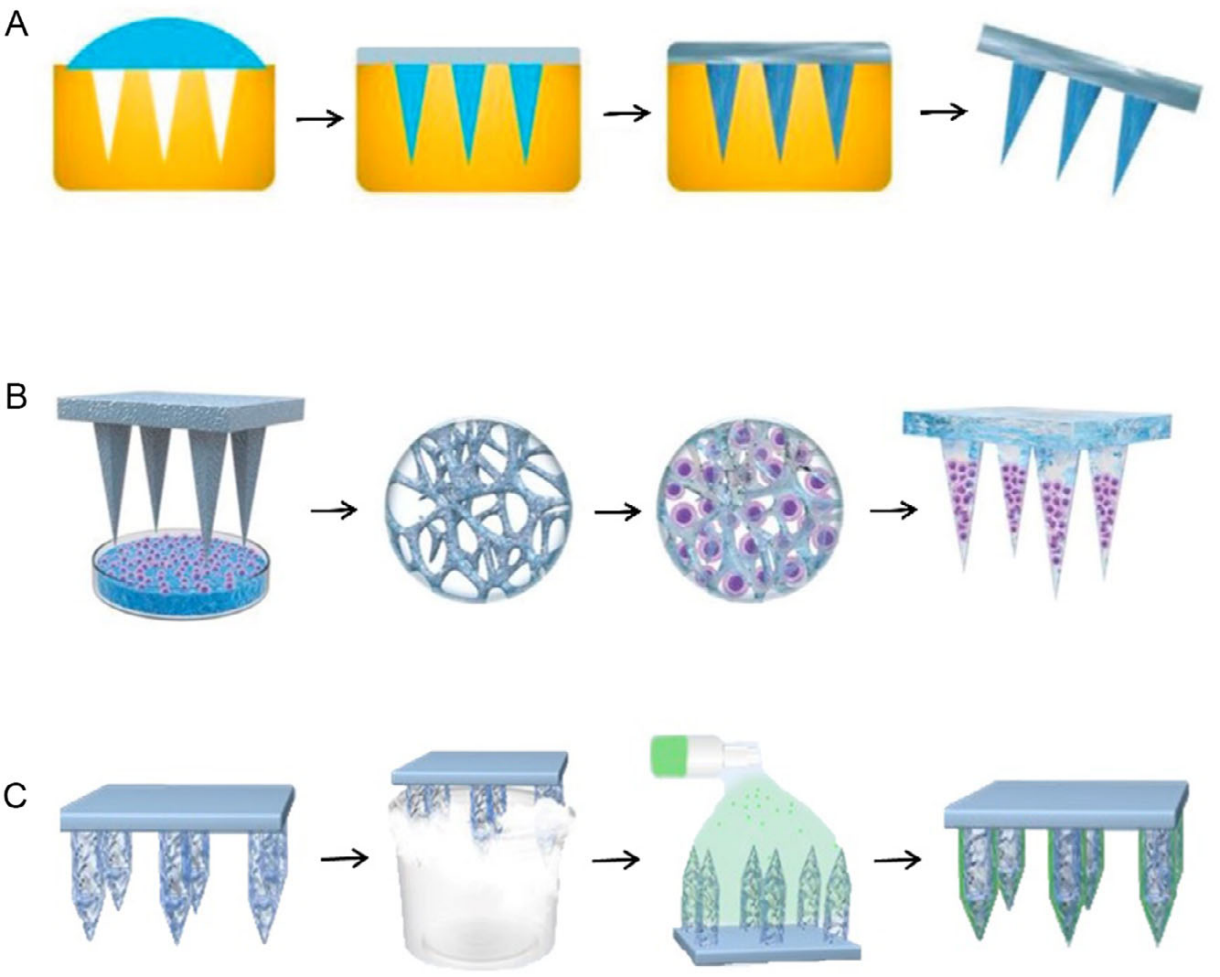
Fabrication methods of cryoMNs. A) The micro‐molding method for cryoMNs fabrication, reproduced with permission,^[^ ^28^ ^]^ copyright 2021, Wiley‐VCH Verlag. B) CryoMNs were fabricated through a dipping and frozen process, reproduced with permission,^[^ ^35^ ^]^ copyright 2024, Springer Nature. C) CryoMNs were fabricated by a sequential spray‐coating and cryogenic freezing protocol, reproduced with permission,^[^ ^36^ ^]^ copyright 2024, John Wiley and Sons Ltd.

The most commonly used technique for fabricating cryoMNs is micro‐molding (**Figure** **2**A), in which MNs molds are fabricated in advance. Stainless‐steel MNs^[^ ^29^ ^]^ and photosensitive resin MNs^[^ ^30^ ^]^ are two main types of positive molds for cryoMNs. Notably, 3D printing technology provides a rapid and customizable method for fabricating positive molds.^[^ ^19^, ^31^, ^32^, ^33^ ^]^ Yang et al. have designed cryoMN positive molds with various shapes through the flexible and controllable static optical projection lithography (SOPL) within 3 seconds.^[^ ^30^, ^34^ ^]^ Negative molds are typically made of polydimethylsiloxane (PDMS)^[^ ^29^ ^]^ or silica gel.^[^ ^30^ ^]^ Once the negative mold is prepared, it is filled with a solution or suspension containing biotherapeutics (often using centrifugation^[^ ^29^ ^]^ or vacuum)^[^ ^30^ ^]^ then frozen to form a needle patch, which is subsequently removed from the mold. Besides, a holder or injector tailored to the MNA can simplify manipulation and ensure rapid penetration.^[^ ^28^, ^30^ ^]^ Another cryoMN fabrication technique (Figure 2B) involves using methacrylated hyaluronic acid (MeHA) to create porous scaffold MNs via lyophilization. These porous MNs are then dipped into a cryogenic medium containing living cells for one minute to fully absorb the solution. The cell‐loaded porous MNs are subsequently transferred to a cryopreservation box and frozen at −80 °C for one day.^[^ ^35^ ^]^ Capillary forces during the wetting process facilitate cells loading into the porous MN scaffold. This scaffold‐assisted fast‐loading method is proposed to minimize the steps between cell harvest and cell delivery. In addition, cryoMNs are fabricated with an iterative spray‐freeze‐drying protocol as illustrated in Figure 2C. The process begins with cryogenic immobilization of hyaluronic acid methacrylate (HAMA) MNs in liquid nitrogen to establish an ultralow temperature matrix.^[^ ^36^ ^]^ This is followed by pneumatic atomization of an exosome‐loaded solution onto the MNs apical surfaces using a precision spray‐coating system. During this step, instantaneous ice crystallization encapsulates the HAMA networks to prevent hydrogel swelling. Subsequent lyophilization in a freeze‐dryer removes residual moisture, with three successive processing cycles ensuring complete exosome integration within the final cryoMNs architecture.

### 2.2 Materials Used in CryoMNs Preparation

In process of cryoMNs preparation, biotherapeutics are encapsulated within matrix materials designed to preserve their bioactivity. These carrier materials should provide structural protection, demonstrate biocompatibility and controlled biodegradability, and maintain the bioactivity retention. Current cryoMNs are classified into two material categories (**Table** **1**). The first category employs pure cryoprotectants including dimethyl sulfoxide (DMSO),^[^ ^29^ ^]^ glycerol,^[^ ^37^ ^]^ trehalose,^[^ ^38^ ^]^ and sucrose.^[^ ^29^ ^]^ The second category utilizes hydrogel‐based matrices such as gelatin methacrylate (GelMA),^[^ ^28^, ^39^ ^]^ matrigel,^[^ ^28^ ^]^ alginate,^[^ ^28^ ^]^ hyaluronic acid (HA),^[^ ^35^, ^40^ ^]^ and methacrylate anhydride‐modified recombinant human collagen.^[^ ^41^ ^]^ These hydrogels are capable of immobilizing bioactive substances (living cells, bacteria, nanoparticles, etc.). Although hydrogel matrices generally exhibit favorable biocompatibility, their structural reinforcement during fabrication often requires photoactivated cross‐linkers. Commonly used initiators, such as lithium phenyl‐2,4,6‐trimethylbenzoylphosphinate (LAP)^[^ ^39^ ^]^ and 2‐hydroxy‐2‐methylpropiophenone (HMPP),^[^ ^28^ ^]^ require stringent residual concentration control to prevent cytotoxicity. Critical evaluation parameters for clinical implementation may encompass three interrelated aspects: matrix degradation kinetics, therapeutic release dynamics, and cryoprotectant specificity (e.g., trehalose for nucleic acid stabilization versus glycerol for microbial viability preservation).

**Table 1:** Summary of cryoMNs in fabrication parameters.^a)^

Reference	Fabrication method	Materials	Freezing protocols	Mechanical strength (needle)	Encapsulated biotherapeutics	Viability	Diseases
Chang, et al.^[^ ^29^ ^]^	Micro‐molding	2.5% DMSO + 100 mM sucrose	−20 °C 4 h, −80 °C 4 h, −196 °C 1 h	0.17 N	DC vaccine	71.4%	Melanoma
Zhang, et al.^[^ ^28^ ^]^	Micro‐molding	Agarose/GelMA/Alginate/Matrigel	−70 °C 24 h	0.8 N	Heparin/erythropoietin/*Bacillus subtilis*	100%	Anticoagulation/anemia/ fungal infections
Cui, et al.^[^ ^37^ ^]^	Micro‐molding	5% Glycerol	4 °C 30 min, −20 °C 4 h, −80 °C 4 h	0.3–0.4 N	*B. bacteriovoru*s	>80%	Eye infection
Chang, et al.^[^ ^42^ ^]^	Micro‐molding	2.5% DMSO + 100 mM sucrose	−20 °C 4 h, −80 °C 4 h, −196 °C	–	DC vaccine + PD‐1 antibody	>85%	Melanoma
Yu, et al.^[^ ^43^ ^]^	Micro‐molding	48 k HA	−80 °C 15 min	0.2‐0.3 N	Comirnaty mRNA lipid nanoparticles	–	COVID‐19
Zheng, et al.^[^ ^39^ ^]^	Micro‐molding	5% GelMA + 5% DMSO CAP	−20 °C 2 h, −80 °C 2 h, −196 °C 1 h	5.175 N	Hair follicle organoid	58.3%–72.5%	Hair loss
Kong, et al.^[^ ^41^ ^]^	Micro‐molding	RHCMA + LAP	−80 °C, 24 h	0.4 N	Nanozymes	–	Eye infection
Yang, et al.^[^ ^44^ ^]^	Micro‐molding	2.5% DMSO + 100 mM sucrose	−20 °C 4 h, −80 °C	0.38 N	Modulated TCVs	53%	Melanoma
Xu, et al.^[^ ^36^ ^]^	Micro‐molding + spray + frozen	HAMA, LAP, zinc acetate, PVA	Liquid nitrogen + freeze‐dryer	0.51 N	Young fibroblast‐derived exosomes	–	Senile wound healing
Li, et al.^[^ ^40^ ^]^	Micro‐molding	HA	−80 °C 24 h	–	HMME + CAT‐biomineralized CCP NFs	–	Melanoma
Zheng, et al.^[^ ^35^ ^]^	Micromolding + lyophilization + frozen	MeHA + 2.5% sodium CMC + 2% DMSO	−80 °C/Liquid nitrogen	–	DC vaccine/MSCs/B16 cells/melanocytes	83.1% –63.4%	Melanoma/vitiligo
Wen, et al.^[^ ^38^ ^]^	Micro‐molding	4% Trehalose	4 °C 40 min, −20 °C 3 h, −80 °C 3 h, −196 °C 1 h	0.18 N	mEXOs‐encapsulated TNF‐α siRNA	–	Rheumatoid arthritis
Shi, et al.^[^ ^45^ ^]^	Micro‐molding	5% Glycerol	−20 °C 4 h, −80 °C 2 h, −196 °C 1 h	0.2–0.3 N	R.r‐Au	70%–90%	Melanoma
Wu, et al.^[^ ^46^ ^]^	Micro‐molding	Plasma‐activated saline	−80 °C 20 min	–	Plasma‐activated saline	–	Psoriasis

a) CAPs, cryopreservation agents; CAT, catalase; CCP NFs, copper phosphate nanoflowers; CMC, carboxymethyl cellulose; DC, dendritic cells; DMSO, dimethyl sulfoxide; GelMA, gelatin methacryloyl; HA, hyaluronic acid; HAMA, hyaluronic acid methacrylate; HMME, hematoporphyrin monomethyl ether; hMSCs, human mesenchymal stem cells; LAP, lithium phenyl‐2,4,6‐trimethylbenzoylphosphinate; MeHA, methacrylated hyaluronic acid; mEXOs, milk‐derived exosomes; PEG, polyethylene glycol; PVA: polyvinyl alcohol; R.r‐Au, nanogold‐engineered *Rhodospirillum rubrum*; RHCMA, methacrylate anhydride‐modified recombinant human collagen; TCVs, whole tumor cell vaccines.

## 3 CryoMNs Applications

### 3.1 CryoMNs‐Mediated Biotherapeutics Delivery for Cancer Immunotherapy

**Figure 3:**
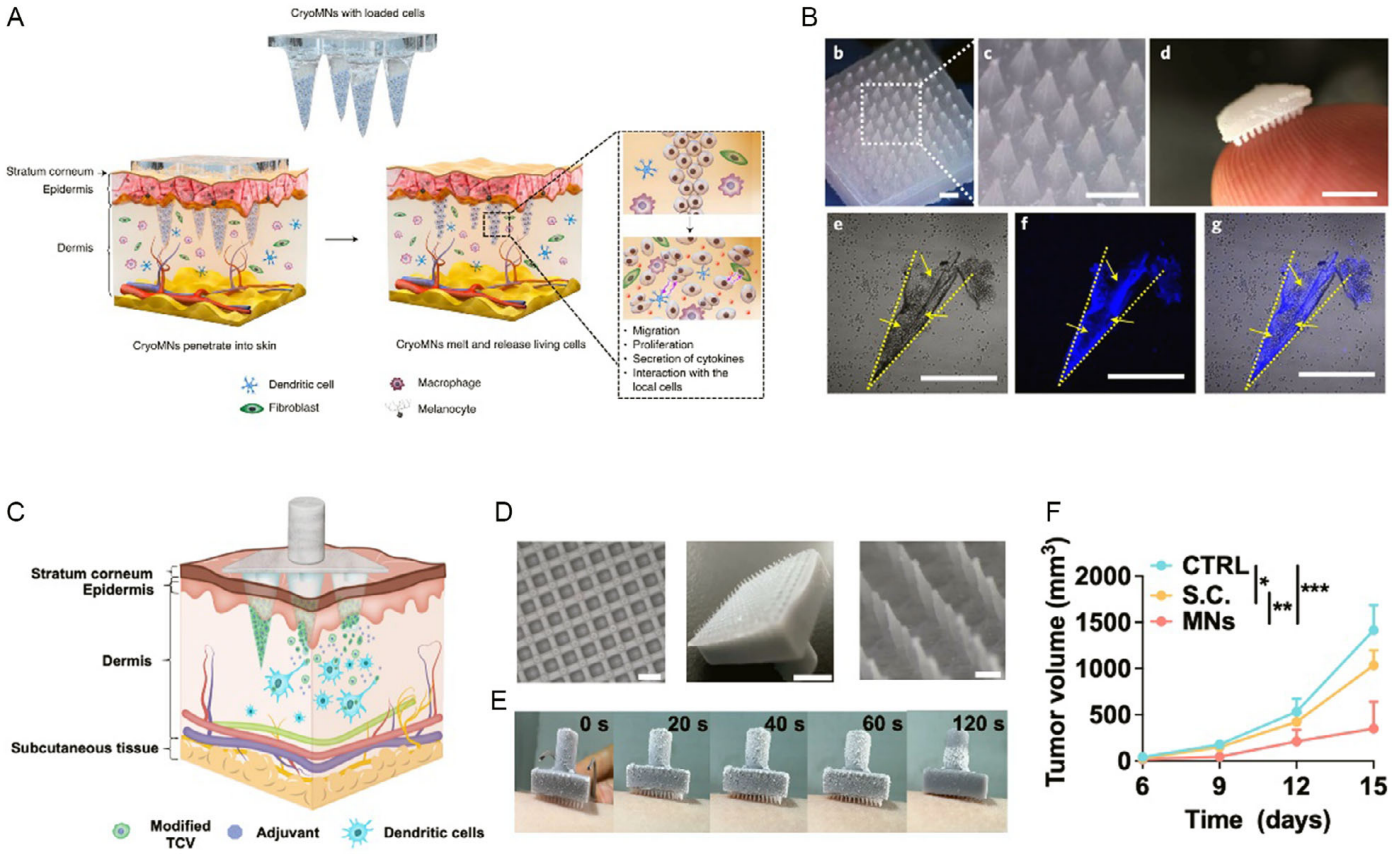
CryoMNs‐mediated cell vaccine for cancer immunotherapy. A) Scheme illustrating the engineering TCV loading cryoMNs penetrated the skin. B) (b) Digital microscopy image of the cryoMNs. Scale bar, 1000 μm. (c) Magnified view of (b). Scale bar, 1000 μm. (d) Photograph of the cryoMNs patch. Scale bar, 5 mm. (e–g) Fluorescent images of cryosections of mouse skin treated with RFP‐HeLa‐cell‐loaded cryoMNs. Scale bar, 200 μm. A and B reproduced with permission,^[^ ^29^ ^]^ copyright 2021, Springer Nature. C) When the ice MNs loaded with TCVs reaching the dermis, rapidly dissolved to release TCVs. The released TCVs secrete adjuvants, attracting dendritic cells and initiating immune response. D) From left to right: bottom view photograph of the holder, scale bar: 1 mm; an ice MN array photograph, scale bar: 5 mm; photograph of an ice MN array under the microscope, scale bar: 500 μm. E) Appearance changes of ice MNs after placement on human skin. F) Tumor growth curves across different groups. C–F reproduced with permission,^[^ ^30^ ^]^ copyright 2024, John Wiley and Sons Ltd.

Cancer remains a formidable challenge to global health, with biotherapeutics emerging as pivotal modalities in oncological interventions.^[^ ^47^, ^48^, ^49^, ^50^, ^51^ ^]^ Vaccine‐based strategies, particularly those leveraging specific immune activation mechanisms, demonstrate significant potential in cancer immunotherapy by eliciting targeted anti‐tumor responses.^[^ ^52^, ^53^, ^54^ ^]^ Compared to traditional subcutaneous vaccination protocols, intradermal delivery systems may exhibit superior immunogenic efficacy. This primarily due to the dense network of antigen‐presenting cells (APCs) within the dermal layer, which enhances antigen capture and presentation.^[^ ^55^ ^]^ Dendritic cells (DCs) are highly efficient in inducing both cellular and humoral immunity. Consequently, efforts have been directed toward enhancing DC vaccines loaded with tumor antigens to initiate anti‐tumor immune responses.^[^ ^56^, ^57^, ^58^ ^]^ In a study conducted by Chang et al.^[^ ^29^, ^42^ ^]^ an ovalbumin (OVA)‐pulsed DCs vaccine was incorporated into cryoMNs for melanoma treatment (**Figure** **3**A). These cryoMNs had a height of ≈900 μm and a base width of ≈350 μm, with stained cells observed inside the needles (Figure 3B). Yang et al. developed ice MNs containing modified TCVs for the sustained release of adjuvants in the dermis for cancer treatment^[^ ^30^ ^]^ (Figure 3C). This cryoMN featured a resin‐based holder, where the connection between the MN bases and the holder's hollow cylinders led to significant drug dosage savings (Figure 3D). When gently applied to skin, the patch rapidly thawed (Figure 3E). In a melanoma model, intradermally delivered antigens by cryoMNs significantly inhibited tumor growth and effectively prevented tumor recurrence without obvious side effects (Figure 3F). Shi et al.^[^ ^45^ ^]^ developed a transdermal therapeutic cryoMNs integrated with nanogold‐engineered *Rhodospirillum rubrum* (R.r‐Au). Under laser irradiation, R.r‐Au enhanced electron transfer by actuating Au nanoparticles into R. R's photosynthetic system. The lactate consumption and hydrogen production were increased to potentiate tumor immune activation.

The application of MNs in cancer treatments has been widely reported.^[^ ^25^, ^59^, ^60^, ^61^ ^]^ Previous studies show that MNs can deliver various biotherapeutics. CryoMNs offer the advantage of preserving the biotherapeutics’ bioactivity for extended periods through the use of low temperatures and cryoprotective agents, potentially making them more suitable for biotherapeutics delivery. However, exposure to low temperatures may influence the efficacy of specific formulations, which should be carefully considered during cryoMNs fabrication. Additionally, cryoMNs can be utilized in treating topical skin tumors. They have the potential to reduce the risk of tumor cell extraction by dissolving rapidly upon reaching skin temperature without removal compared to other types of MNs.

### 3.2 CryoMNs‐Mediated Biotherapeutics Delivery for Infectious Diseases Prevention and Treatment

**Figure 4:**
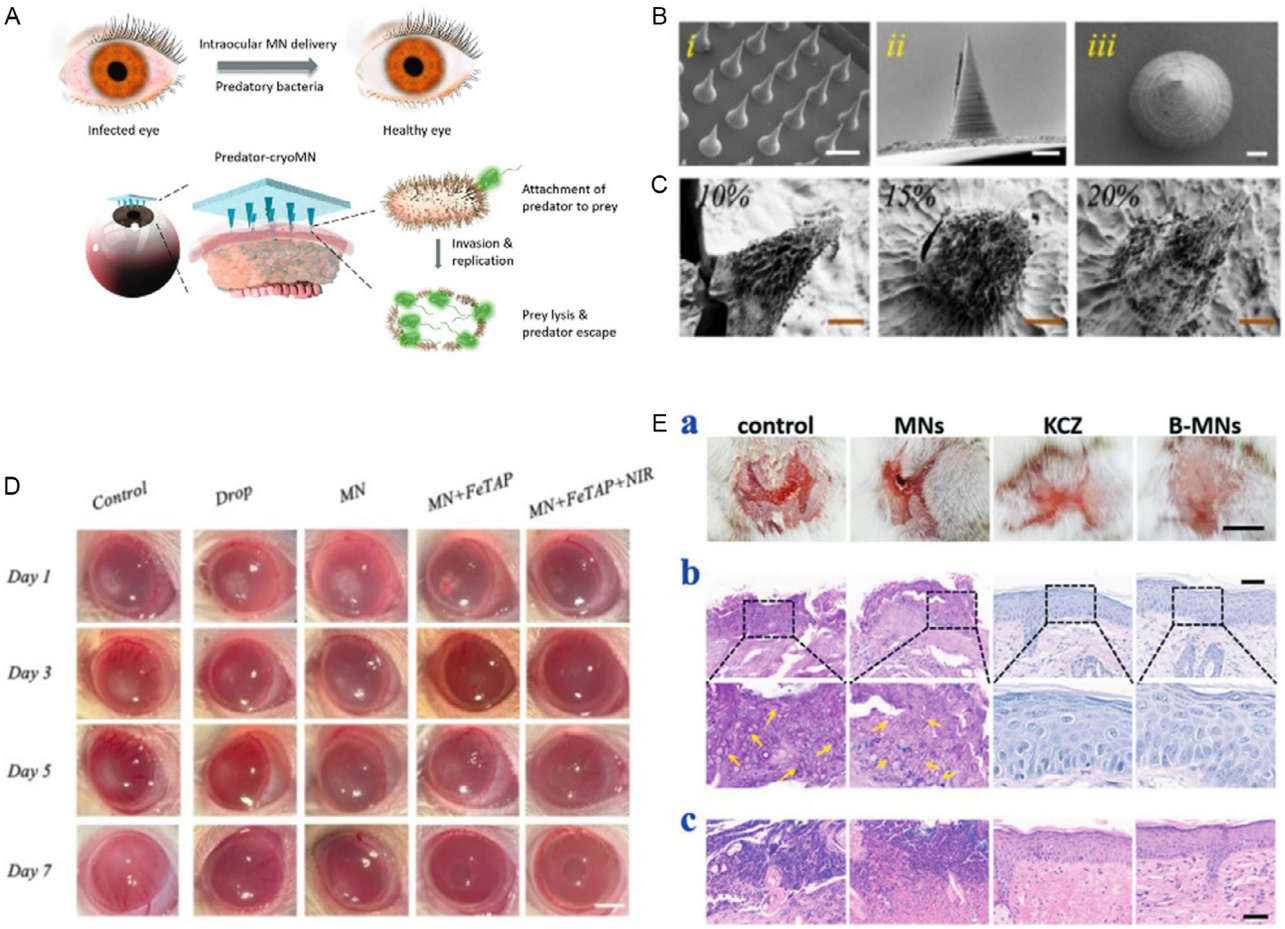
CryoMNs‐mediated biotherapeutics for eye infection treatment. A) Illustration of cryoMNs for ocular delivery of predatory bacteria in treating eye infection, reproduced with permission,^[^ ^37^ ^]^ copyright 2021, Wiley‐VCH Verlag. B) SEM images of the FR‐MNs with different side views. Scale bars, (i) 500 μm, (ii) 100 μm, (iii) 30 μm. C) SEM images of the freeze‐drying FR‐MNs fabricated with different RHCMA concentrations. Scale bar, 50 μm. D) Representative photographs of infected corneas treated with drops, FR‐MNs, FeTAP + FR‐MNs, and FeTAP + FR‐MNs + NIR for different times. (B,D) were reproduced with permission,^[^ ^41^ ^]^ copyright 2023, Elsevier. E) (a) Representative photos of back skins of the mice in the control group, the MNs group, the KCZ group, and the B‐MNs group on Day 13. (b) PAS staining of mouse back skins in different groups on Day 13. (c) H&E staining of mouse back skins in different groups on Day 13, reproduced with permission,^[^ ^28^ ^]^ copyright 2021, Wiley‐VCH Verlag.

Infectious diseases are defined as illnesses caused by specific infectious agents or their toxic products.^[^ ^62^ ^]^ These diseases are primarily related to three classes of pathogenic microorganisms: bacteria, viruses, and fungi.^[^ ^63^ ^]^ Vaccines are the most cost‐effective biomedical intervention for disease prevention.^[^ ^64^, ^65^ ^]^ The emergence of mRNA vaccine platforms has revolutionized prophylactic strategies by enabling rapid development cycles, lyophilized formulations, and scalable production capacities.^[^ ^66^, ^67^ ^]^ However, most mRNA vaccines are unstable at room temperature and must be stored and transported in low‐temperature freezers. To address this issue, Yu et al.^[^ ^43^ ^]^ used liposomes‐based cryoMNs for mRNA vaccine intradermal delivery to prevent COVID‐19. CryoMNs successfully induced both neutralizing antibodies and T‐cell responses. In addition to preventing system infectious diseases, cryoMNs are also applied in the treatment of local infections. Due to the presence of biological barriers or tight connections in certain tissues, drug penetration is often limited, resulting in low bioavailability. To treat bacterial eye infections, researchers have developed a strategy using predatory bacteria by introducing cryoMNs for the ocular delivery of *Bdellovibrio bacteriovorus* ^[^ ^37^ ^]^ (**Figure** **4**A). Utilizing the protective effect of low temperatures on enzyme activity, Kong et al.^[^ ^41^ ^]^ proposed nanozyme‐loaded cryoMNs that exhibited superior therapeutic potential in a rat eye infection model (Figure 4B–D). In addition, since *Bacillus subtilis* could secrete antimicrobial agents and compete with other colonies for nutrients and space, it had broad‐spectrum antimicrobial properties against bacteria and fungi. Zhang et al.^[^ ^25^ ^]^ have developed *B. subtilis*‐loaded cryoMNs for skin fungal infections treatment (Figure 4E). Other infectious diseases are also reported by MNs mediated treatment.^[^ ^68^, ^69^ ^]^ Furthermore, MNs mediated influenza and polio vaccines have been intradermally administered in clinical trial and achieved similar immune responses compared to those of intramuscular injections.^[^ ^70^, ^71^, ^72^ ^]^ CryoMNs may be particularly suitable for the long‐term stabilization of mRNA vaccine. As cryoMN technology undergoes progressive optimization and scaled production through standardized protocols, it is anticipated to facilitate clinical translation in future therapeutic applications.

### 3.3 CryoMNs‐Mediated Biotherapeutics Delivery for Autoimmune Diseases Therapy

**Figure 5:**
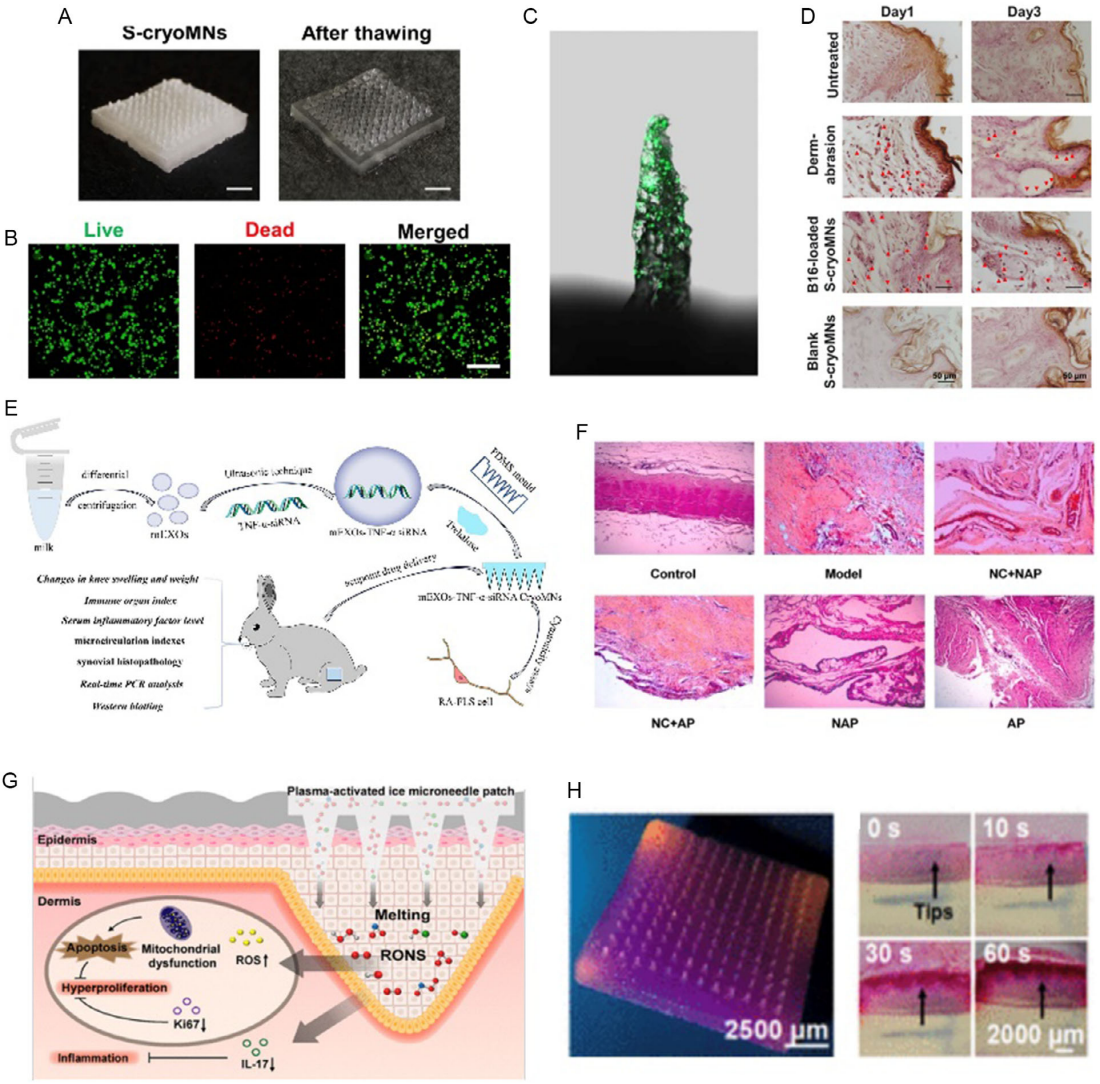
CryoMNs‐mediated biotherapeutics for autoimmune diseases therapy. A) Digital images of cryoMNs before and after thawing. Scale bar: 2 mm. B) Live/dead staining of A375 cells after their release from cryoMNs. Scale bar: 200 μm. C) Confocal images of cryoMNs loaded with A375 labeled with CellTracker (green). Scale bar: 200 μm. D) Melanin expression of the transplanted B16 cells using dermabrasion, blank or B16‐loaded cryoMNs 1 and 3 days after application. Melanin was stained with Masson‐Fontana stain and is indicated by the red arrow. (A–D) were reproduced with permission,^[^ ^35^ ^]^ copyright 2024, Springer Nature. E) CryoMNs combined with exosomes and acupoints drug delivery for the treatment of RA. F) Effect of TNF‐α siRNA cryoMNs on synovial histopathology in rabbits. cryoMNs, cryomicroneedle; TNF‐α, tumor necrosis factor‐α. (E,F) were reproduced with permission,^[^ ^38^ ^]^ copyright 2024, Elsevier. G) PA‐IMNs loaded with multiple RONS are designed for local transdermal delivery to treat psoriasis as an alternative to direct CAP irradiation treatment. H) Photograph of the PA‐IMN patch loaded with the Griess reagent and release of NO_2_– from the PA‐IMN patch in the chromogenic gelatin model. (G) and (H) were reproduced with permission,^[^ ^46^ ^]^ copyright 2024, American Chemical Society.

Autoimmune diseases comprise a diverse spectrum of disorders characterized by aberrant immune responses of B and T lymphocytes against self‐tissues.^[^ ^73^ ^]^ Current therapeutic strategies predominantly depend on biologics that selectively target defined pro‐inflammatory signaling cascades or immune effector mechanisms.^[^ ^74^, ^75^ ^]^ Zheng et al.^[^ ^35^ ^]^ fabricated a porous MN scaffold using MeHA as a proof of concept for vitiligo treatment. The MN scaffold was sequentially processed by dipping into a melanoma cell (secreting melanin) suspension followed by cryogenic solidification (**Figure** **5**A). This cryoMN platform achieved a cell loading density of 240 cells needle^−1^ at an optimized concentration of 1.0 × 10^7^ cells mL^−1^ (Figure 5B,C). When applied to mouse skin, the cryoMNs loaded with melanoma cells successfully delivered cells to the epidermal basal layer and around hair follicles. They remained viable and capable of melanin production for at least three days post‐application (Figure 5D). Composed solely of water and modified melanin, this cryoMN structure shows significant potential for clinical application. CryoMNs have also shown promise in rheumatoid arthritis and psoriasis treatment. Wen et al.^[^ ^38^ ^]^ developed cryoMNs encapsulating TNF‐α siRNA within milk‐derived exosomes (mEXOs), and its efficacy was tested through local transdermal delivery at acupuncture points for rheumatoid arthritis (Figure 5E). In vivo pharmacodynamics studies found that general conditions, microcirculation indices, synovial histopathology, and expression of related proteins in the synovial tissue of rheumatoid arthritis rabbits were effectively alleviated by mEXOs‐TNF‐α siRNA cryoMNs (Figure 5F). Wu et al.^[^ ^46^ ^]^ introduced plasma‐activated ice MNs (PA‐IMNs) for psoriasis treatment, which facilitate the transdermal delivery of reactive oxygen and nitrogen species (RONS) (Figure 5G). Prepared from saline activated by plasma discharge in NOx and O_3_ modes, the PA‐IMN array produces high‐valency RONS. The frozen PA‐IMNs have robust mechanical strength, enabling penetration of the thickened stratum corneum and delivery of RONS to deeper tissue layers (Figure 5H). Transdermal treatment confirms that PA‐IMNs produce significant anti‐inflammatory and therapeutic actions for imiquimod (IMQ)‐induced psoriasis‐like dermatitis in mice, by inhibiting the release of associated inflammatory factors without evident systemic toxicity.

Moreover, recent studies have highlighted the potential of regulatory T (Treg) cells and CAR‐T cells in autoimmune disease treatment.^[^ ^76^, ^77^, ^78^, ^79^, ^80^ ^]^ Local delivery of Treg cells via MNs has been shown to significantly reduce psoriasis inflammation in a mouse model, providing an alternative to intravenous or intradermal injections.^[^ ^81^ ^]^ These strategies are equally applicable to cryoMNs for the delivery of Tregs or CAR‐T cells. However, maintaining long‐term cell viability and optimizing cell loading capacity remain challenges for both cryoMNs and other MN‐based therapies.

### 3.4 CryoMNs‐Mediated Biotherapeutics Delivery for Tissue Regeneration

**Figure 6:**
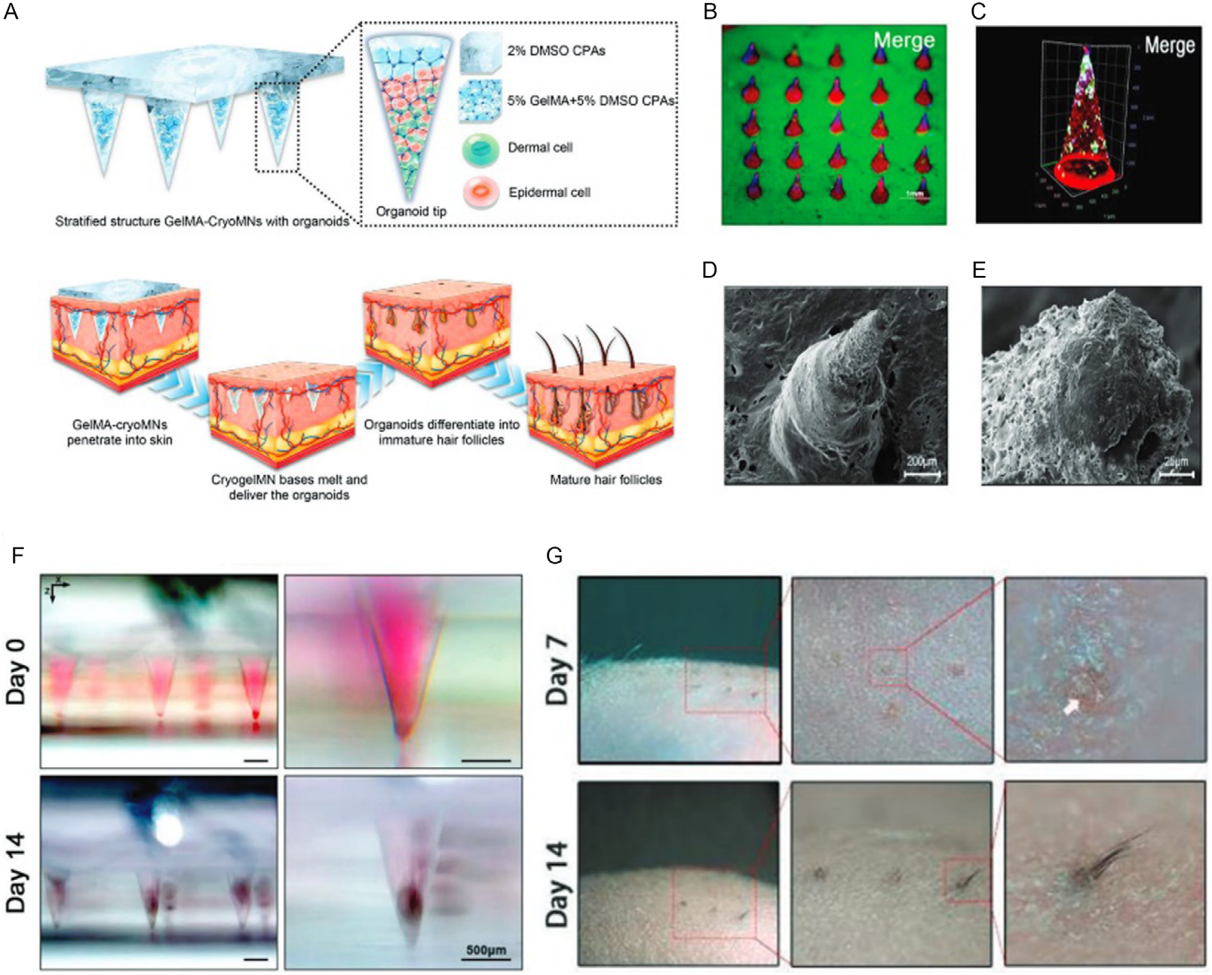
CryoMNs‐mediated biotherapeutics for hair regeneration. A) Schematic illustration of the transdermal delivery of organoids with GelMA‐cryoMNs and hair follicle differentiation. B) Photograph of the cryoMNs array with DAPI organoids (blue), rhodamine B‐tip (red), and FITC‐base (green). Scale bars, 1 mm. C) 3D reconstruction images of cell distribution in single tip with DiO‐DCs (green), DAPI‐ECs (blue), and rhodamine B‐tip (red) under LSCM. D,E) Porous structure of organoids‐loaded GelMA‐cryoMNs after lyophilization under SEM. F) Differentiation of HF organoid constructed with DCs and ECs delivered by GelMA‐cryoMNs in vitro culture. Images of cells self‐assembly and differentiation process under stereo microscopy in front view at Day 0 and Day 14. Scale bars: 500 μm. G) Lateral and detailed view of reconstructed hairs with GelMA‐croMNs at Day 7 and Day 14. (A–G) were reproduced with permission,^[^ ^39^ ^]^ copyright 2023, Wiley‐VCH Verlag.

Tissue regeneration or repair refers to the restoration of damaged tissue to its normal state with full functionality.^[^ ^82^ ^]^ Hair follicle (HF) regeneration is emerging as a promising therapeutic strategy to reconstruct functional HFs for addressing alopecia.^[^ ^83^ ^]^ Organoids have become a pivotal focus in the field of HF tissue engineering and regeneration.^[^ ^84^ ^]^ Zheng et al.^[^ ^39^ ^]^ developed a GelMA‐based cryopreservation hydrogel suitable for both the fabrication of cryoMNs and the maintenance of cell viability for hair regeneration (**Figure** **6**A). These cryoMNs have organoid‐loaded tips, while the MN base is composed solely of cryoprotective medium (Figure 6B–E). The GelMA‐embedded organoids delivered by the GelMA‐cryoMNs underwent a developmental process within the skin, ultimately leading to the reconstruction of mature HFs. On Day 14 of in vitro culture, pigmentation of the sphere‐like cysts deepened, and hair bulbs protruding from the cyst surface were observed (Figure 6F). A lateral view, along with further magnification of the GelMA‐cryoMN implantation sites, revealed the presence of black‐brown follicle‐like structures within the superficial layer of the mouse skin on Day 7 (Figure 6G). On Day 14, several parallel tufts of black hairs, aligned with the MN array, had broken through the skin surface vertically (Figure 6G). These newly formed hairs were clearly distinguishable from the native small white hairs, which grew in a disordered manner in nude mice.

Meanwhile, MNs can also provide effective regeneration for other tissues through the delivery of biotherapeutics, especially for living cell delivery. For skin wounds, mesenchymal stem cells^[^ ^85^ ^]^ or adipose‐derived stem cells^[^ ^86^ ^]^ are reported to promote skin regeneration. For myocardial infarction, induced pluripotent stem cells^[^ ^87^ ^]^ or cardiac stromal cells^[^ ^88^ ^]^ have been used to repair cardiac. Additionally, umbilical artery‐derived perivascular stem cells and antioxidant nanozymes have been applied for endometrial repair in damaged uterus.^[^ ^89^ ^]^ Similarly, MNs have been used to load islet β‐cells directly to the skin for insulin secretion in diabetes, bypassing traditional transplantation. CryoMNs that can deliver living cells and organoids have comparable mechanical properties with other MNs, expanding their potential applications in tissue repair.

## 4 Future Perspectives and Conclusions

CryoMNs represent an innovative therapeutic platform that synergizes cryopreservation technology with transdermal drug delivery, offering transformative potential for biomedical applications. Precision personalized therapy could be revolutionized by leveraging cryopreservation to stably encapsulate and preserve sensitive biologics. This capability enables spatiotemporally controlled drug release, positioning cryoMNs as promising tools for regenerative medicine applications. Moreover, technological convergence is reshaping MN design through advanced manufacturing techniques. For instance, 3D printing allows architectural customization to optimize drug‐loading capacity and release kinetics. Integration with artificial intelligence‐driven drug formulation design and stimuli‐responsive materials further enables intelligent, on‐demand therapeutic activation. In addition, the clinical applications of cryoMNs are expanding to address systemic diseases, including novel vaccine delivery systems and digital health integration for infectious disease monitoring.

However, the clinical translation of cryoMNs is limited due to several significant challenges. A critical barrier lies in the rapid melting tendency of cryoMNs under ambient conditions, which compromises structural integrity within minutes and triggers premature drug leakage. This instability necessitates stringent cold‐chain storage requirements. The devices may become vulnerable to mechanical degradation during repeated freeze‐thaw cycles, ultimately risking sterility breaches. Another key obstacle involves material and structural instability, where cryogenic processing may induce polymer matrix crystallization or drug‐carrier phase separation. It can lead to inconsistent mechanical performance, evidenced by elevated insertion failure rates and heterogeneous drug distribution. Scalability limitations further complicate fabricating, as balancing cryogenic parameters with production efficiency remains elusive. Rapid cooling weakens MNs integrity, whereas slower protocols causes protein denaturation. Additionally, uncertainties still persist regarding in vivo behavior, particularly poorly characterized degradation kinetics and unresolved biocompatibility risks.

To address these challenges, multidisciplinary strategies are emerging. Material innovations focus on hybrid matrices that integrate cryoprotectants with structural reinforcement agents. The nanofiber self‐assembly techniques can enhance mechanical robustness and bioactivity preservation. Process optimization leverages microfluidic chip‐assisted directional freezing for high‐throughput fabrication supplemented by machine learning algorithms. The optimal cryopreservation parameters can be predicted, and the experimental iterations can be minimized. Advanced evaluation systems with embedded fluorescent nano‐sensors and organ‐on‐a‐chip models enable real‐time monitoring of drug release profiles and tissue‐specific permeation dynamics. Concurrently, collaborative translational efforts aim to establish cross‐sector consortia to standardize fabricating workflows and accelerate regulatory approval through microdose clinical trials. Collectively, these advances could unlock the full potential of cryoMNs as a novel drug delivery platform, contingent upon resolving critical gaps in stability, scalability, and biological predictability.

In summary, the use of cryoMNs for delivering biotherapeutics shows promise in the field of biotherapy. Further research is needed to optimize the fabrication process and explore the pharmacokinetics mediated by cryoMNs, laying a solid foundation for subsequent clinical trials. CryoMNs that can maintain bioactivity while being easily self‐operable by patients have the potential to change the future of biotherapeutics delivery administration.

## Conflict of Interest

The authors declare no conflict of interest.

## Author Contributions

**Chunli Yang**: investigation, methodology, validation, visualization, data curation, writing—original draft. **Li Zhang**: visualization, formal analysis, software. **Angxi Zhou**: methodology, validation. **Siyi Wang**: methodology, validation. **Ya Ren**: methodology. **Maya Xiang**: methodology. **Run Tian**: methodology. **Yang Yu**: methodology. **Rong Li**: methodology. **Maling Gou**: conceptualization, funding acquisition, project administration, resources, supervision, writing—review and editing.
